# Supplementary material for: Effect of drainage layers on water retention of potting media in containers
Source: PLoS One. 2025 Feb 25;20(2):e0318716. doi: 10.1371/journal.pone.0318716 (PMC11856292; doi:10.1371/journal.pone.0318716)
Supplement: S1 Appendix — (PDF) [file pone.0318716.s001.pdf]

Table 1. ANOVA for CPB medium by combined drainage layer type

Cases	Sum of Squares	df	Mean Square	F	p
Drainage layer type	0.089	8	0.011	26.660	$< .001$
Residuals	0.034	81	4.181×10^{-4}		

Note. Type III Sum of Squares

Table 2. ANOVA for CPB medium by drainage layer substrate

Cases	Sum of Squares	df	Mean Square	F	p
Drainage layer substrate	0.082	4	0.021	42.834	$< .001$
Residuals	0.041	85	4.800×10^{-4}		

Note. Type III Sum of Squares

Table 3. ANOVA for CPB medium by drainage layer depth

Cases	Sum of Squares	df	Mean Square	F	p
Drainage layer depth	0.073	2	0.037	64.434	$< .001$
Residuals	0.050	87	5.700×10^{-4}		

Note. Type III Sum of Squares

Table 4. ANOVA for CV medium by combined drainage layer type

Cases	Sum of Squares	df	Mean Square	F	p
Drainage layer type	0.133	8	0.017	17.843	< .001
Residuals	0.074	80	9.284×10^{-4}		

Note. Type III Sum of Squares

Table 5. ANOVA for CV medium by drainage layer substrate

Cases	Sum of Squares	df	Mean Square	F	p
Drainage layer substrate	0.057	4	0.014	7.978	< .001
Residuals	0.150	84	0.002		

Note. Type III Sum of Squares

Table 6. ANOVA for CV medium by drainage layer depth

Cases	Sum of Squares	df	Mean Square	F	p
Drainage layer depth	0.081	2	0.041	27.835	< .001
Residuals	0.126	86	0.001		

Note. Type III Sum of Squares

Table 7. ANOVA for JI medium by combined drainage layer type

Cases	Sum of Squares	df	Mean Square	F	p
Drainage layer type	0.067	8	0.008	9.882	$< .001$
Residuals	0.069	81	8.526×10^{-4}		

Note. Type III Sum of Squares

Table 8. ANOVA for JI medium by drainage layer substrate

Cases	Sum of Squares	df	Mean Square	F	p
Drainage layer substrate	0.048	4	0.012	11.514	$< .001$
Residuals	0.089	85	0.001		

Note. Type III Sum of Squares

Table 9. ANOVA for JI medium by drainage layer depth

Cases	Sum of Squares	df	Mean Square	F	p
Drainage layer depth	0.009	2	0.004	3.011	0.054
Residuals	0.128	87	0.001		

Note. Type III Sum of Squares
